# Transcranial Doppler Ultrasonographic Evaluation of Cerebrovascular Abnormalities in Children With Acute Bacterial Meningitis

**DOI:** 10.3389/fneur.2020.558857

**Published:** 2021-02-12

**Authors:** Yudy Fonseca, Taty Tshimanga, Stephen Ray, Helen Malhotra, Jean Pongo, Joseph Bodi Mabiala, Montfort Bernard Gushu, Tusekile Phiri, Bertha Mekiseni Chikaonda, Davin Ambitapio Musungufu, Mananu Uchama, Nicole Fortier O'Brien

**Affiliations:** ^1^Division of Critical Care Medicine, Department of Pediatrics, University of Maryland, Baltimore, MD, United States; ^2^Department of Pediatrics, University of Kinshasa, Kinshasa, Democratic Republic of Congo; ^3^Malawi Liverpool Wellcome Trust Clinical Research Programme, Paediatric Registrar & Wellcome Trust Clinical Fellow, Blantyre, Malawi; ^4^Department of Behavioral Neuroscience, Northeastern University, Boston, MA, United States; ^5^Department of Medicine, Universite des Sciences et des Technologie de Lodja (USTL), Lodja, Democratic Republic of Congo; ^6^Department of Pediatrics, Queen Elizabeth Central Hospital, Blantyre Malaria Project, Blantyre, Malawi; ^7^L'Hopital Generale de Reference de Nyankunde, Nyankunde, Democratic Republic of Congo; ^8^Division of Critical Care Medicine, Department of Pediatrics, Nationwide Children's Hospital, The Ohio State University, Columbus, OH, United States

**Keywords:** meningitis, transcranial Doppler ultrasound, neurovascular, pediatrics, bacterial

## Abstract

**Introduction:** Bacterial meningitis (BM) is a global public health concern that results in significant morbidity and mortality. Cerebral arterial narrowing contributes to stroke in BM and may be amenable to intervention. However, it is difficult to diagnose in resource-limited settings where the disease is common.

**Methods:** This was a prospective observational study from September 2015 to December 2019 in sub-Saharan Africa. Children 1 month−18 years of age with neutrophilic pleocytosis or a bacterial pathogen identified in the cerebrospinal fluid were enrolled. Transcranial Doppler ultrasound (TCD) of the middle cerebral arteries was performed daily with the aim to identify flow abnormalities consistent with vascular narrowing.

**Results:** Forty-seven patients were analyzed. The majority had *Streptococcus pneumoniae* (36%) or *Neisseria meningitides* (36%) meningitis. Admission TCD was normal in 10 (21%). High flow with a normal pulsatility index (PI) was seen in 20 (43%) and high flow with a low PI was identified in 7 (15%). Ten (21%) had low flow. All children with a normal TCD had a good outcome. Patients with a high-risk TCD flow pattern (high flow/low PI or low flow) were more likely to have a poor outcome (82 vs. 38%, *p* = 0.001).

**Conclusions:** Abnormal TCD flow patterns were common in children with BM and identified those at high risk of poor neurological outcome.

Bacterial meningitis (BM) results in significant morbidity and mortality worldwide (1). Over 1 million individuals are affected annually, with the highest burden of disease occurring in children in sub-Saharan Africa (2). Case fatality rates range from 8 to 15% [3]. In the absence of early intervention, mortality approaches 50% (3). Half of survivors are left with permanent neurologic sequelae such as cognitive impairment, spastic quadriplegia, and/or seizures (4–8). These poor outcomes are largely attributable to meningitis-related intracranial complications (7–11).

Ischemic stroke is one such event occurring in 20–25% of patients with BM (11–13). The predominant pathological change to the neurovasculature on angiogram in these patients is cerebral arterial narrowing (10, 13). While the underlying etiology for this narrowing is still not completely understood, vasculitis, vasospasm, intra-arterial thrombosis, and external compression of vessels by purulent material in the subarachnoid space have all been implicated (8–13).

The early identification of poor or deteriorating cerebral hemodynamics secondary to vascular narrowing in patients with BM may create a therapeutic window in which measures to increase cerebral perfusion pressure (CPP) are undertaken. The goal of CPP augmentation in this situation would be to overcome the increased resistance of the cerebral arteries and provide sufficient substrate delivery to meet metabolic demand, thus reducing secondary ischemic injury (14, 15). When CPP-targeted treatment was provided indiscriminately to a group of children with CNS infection in India, those with BM had a 90-day mortality reduction from 41 to 10% (*p* < 0.01) (16). However, the empiric augmentation of CPP requires significant resources, is likely not necessary for some patients, and may be dangerous to others. Thus, identifying patients with compromised cerebral hemodynamics due to vascular narrowing that are most likely to benefit from such therapy may be beneficial.

Advanced neuroimaging approaches commonly used to diagnose pathophysiological changes to the neurovasculature in developed countries are not widely available in the regions of the world that are most heavily impacted by BM. Transcranial Doppler Ultrasound (TCD) is a portable, non-invasive, inexpensive tool that evaluates cerebral blood flow velocities (CBFVs) (17–19). It has been used to identify disease-related arterial narrowing in both adults and children with BM in Europe and the USA (20–26). Increases in CBFVs in these studies were associated with lower admission Glasgow Coma Scores (GCS), stroke, and poor long-term outcome.

There are no reports on TCD findings or associations with outcomes in children with BM in sub-Saharan Africa. Given the high burden of disease, the differing causative pathogens, and the comorbidities in this population, we performed this prospective, observational study. CBFVs become elevated and then profoundly reduced as the degree of arterial narrowing progresses in other disease states (27, 28). We therefore hypothesized that TCD-derived CBFVs could be categorized as normal, increased, then significantly reduced in children with BM. We also hypothesized that high-risk flow patterns suggestive of severe arterial narrowing would be associated with poor outcomes.

## Materials and Methods

### Study Population

The study was performed from September 2015 to December 2019 at Kalembe Lembe Children's Hospital in the Democratic Republic of the Congo (DRC), L'Hopital General de Reference de Lodja in the DRC, L'Hopital General de Reference de Nyankunde in the DRC, and Queen Elizabeth Central Hospital (QECH) in Blantyre, Malawi. Ethics approval was granted by involved institutions and parents or guardians signed written consent before enrollment. Children 1 month−18 years of age were approached for enrollment when diagnosed with BM according to the following criteria: (1) Gram stain or culture of the cerebrospinal fluid (CSF) positive for a bacterial organism OR (2) CSF with ≥100 leukocytes/mm^3^ with >60% neutrophils (culture-negative meningitis).

Children with sickle cell anemia or cerebral malaria were excluded given the high frequency of abnormal TCD findings in these children. Given the unknown impact of severe malnutrition or advanced HIV disease on CBFVs, children diagnosed with one of these conditions (mid-upper arm circumference <11.5 cm or known HIV positivity with severe wasting) were excluded.

Demographic data, vital signs, and Blantyre Coma Score (BCS) at presentation were collected. Finger-prick samples were analyzed to determine blood glucose and lactate concentration (EKF Biosen glucose and lactate analyzer, Penarth, England). Venous blood was drawn to obtain a complete blood count (Coulter Counter; Beckman Coulter, Indianapolis, IN). CSF was analyzed using the standard methods for cell count, protein, Gram stain, and culture.

### Transcranial Doppler Ultrasound Examinations

#### Primary TCD Measurements

TCD was performed using a commercially available TCD unit (SonaraTek Digital TCD, CareFusion, Middleton, WI or Lucid TCD, Neural Analytics, Los Angeles, CA). Daily TCD was performed through hospital day 8, discharge, or death, whichever occurred first. In patients with abnormal TCD findings at day 8, weekly TCD was performed until discharge. Middle cerebral arteries (MCAs) and extracranial internal carotid arteries (EC-ICAs) were insonated at 2-mm intervals using previously described methods (18). Systolic (Vs), diastolic (Vd), and mean flow velocities (Vm) were recorded.

#### Ancillary TCD Measurements

Pulsatility index (PI = (Vs – Vd/Vm)), a marker of downstream cerebrovascular resistance (CVR), was automatically calculated by the TCD unit at each depth. As distal CVR decreases, diastolic flow rises and the PI decreases. As distal CVR increases, diastolic flow falls and the PI increases.

To differentiate causes of high measured CBFVs, the Lindegaard ratio (LR = MCA Vm/Ex-ICA Vm) was calculated (19). A LR <3 was considered to represent hyperemia whereas a LR >3 was considered to represent vascular narrowing, with progressive increases in the LR considered to be suggestive of worsening narrowing.

Autoregulation is the capacity to maintain constant cerebral blood flow over a wide range of blood pressures. The transient hyperemic response ratio (THRR = average Vm of 2 beats after a 10-s carotid compression/average Vm of 5 beats before compression) was used to interrogate cerebral autoregulation (29). A THRR < 1.1 was considered to represent impaired autoregulation and ≥1.1 intact autoregulation (29).

#### Diagnostic Criteria

The definitions used to categorize study patients were:

1. Normal flow—Vs, Vd, and Vm within 2SD of age normative value (20).
2. High flow—Vs, Vm >2SD above age normative value.
  (a) Normal PI—PI ≥0.6 and ≤ 1.3.
  (b) Abnormal PI—PI ≤ 0.5 (low PI) or >1.3 (high PI).
3. Low flow—Vs and Vm <2SD below age normative value.
  (a) Normal PI—PI ≥0.6 and ≤ 1.3.
  (b) Abnormal PI—PI ≤ 0.5 (low PI) or >1.3 (high PI).

**Figure 1:**
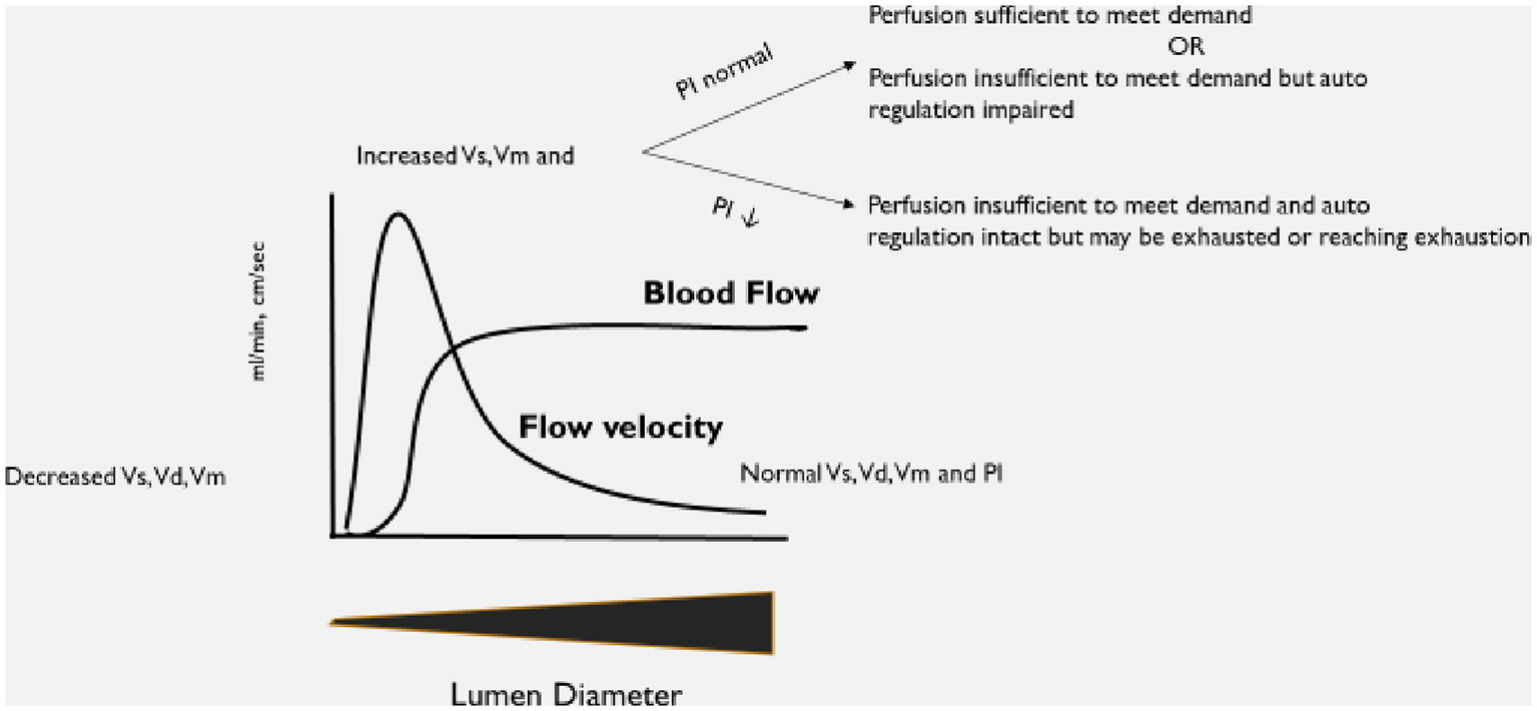
Explanation of expected changes to transcranial Doppler ultrasound (TCD) flow velocities and patterns as vessel lumen diameter changes.

An explanation of the basis for the selected diagnostic criteria is in Figure 1. As this was an observational study, no changes to the patient's care were undertaken based on the results of the TCD examination.

#### Outcomes

Pediatric cerebral performance category (PCPC) scoring was performed at hospital discharge (30, 31). Children with a PCPC of 1 or 2 were considered to have a good outcome while those with a PCPC of 3–6 were considered to have a poor outcome.

#### Study Oversight

The study was approved by the ethics committee at the University of Kinshasa, School of Public Health, DRC. The study was also approved by the ethics committee at the University of Malawi College of Medicine Research Ethics Committee (COMREC). All subjects' guardians provided informed consent before enrollment.

#### Statistical Analysis

Primary results are restricted to the first observation per patient. Categorical variables are summarized using frequencies and percentages, normally distributed continuous variables are summarized using means with SDs, and non-normally distributed continuous variables are summarized using medians with interquartile range (IQR). Comparisons by organism and pattern were evaluated using χ^2^ or Fisher's exact test, one-way ANOVA with a Tukey correction for multiple comparisons, or Kruskal–Wallis tests with Dwass–Critchlow–Flinger–Steel corrections for multiple comparisons. Comparisons between patterns were evaluated using χ^2^ or Fisher's exact tests, two-sample *t*-tests, or Wilcoxon rank sum tests. As a sensitivity analysis, for variables with some longitudinal collection, mixed-effects models with random subject effects were run to evaluate comparisons using all available data, while accounting for within-patient correlation and differences in number of measurements. Given the sample size, multivariate analysis was not performed. All analyses were conducted using SAS 9.4 (SAS Institute, Cary, NC).

## Results

Forty-seven patients were included. Demographics, vital signs, and laboratory variables for participants are in Table 1. The majority of the patients had *Streptococcus pneumoniae* (n = 17, 36%) or *Neisseria meningitides* (*n* = 17, 36%) meningitis. Fewer were diagnosed with *Salmonella* sp. (*n* = 6, 13%) or culture-negative meningitis (*n* = 5, 11%). One child was infected with *Staphylococcus aureus* (2%) and one with *Escherichia coli* (2%). Children with meningococcal meningitis were more likely to have convulsions than children infected with other organisms (*n* = 14/17 (82%) for *N. meningitides* vs. *n* = 10/17 (59%) for *S. pneumoniae* vs. *n* = 1/6 (17%) for *Salmonella* sp., vs. *n* = 3/5 (60%) for culture-negative meningitis, *p* = 0.03). CSF cell count was significantly different depending on infecting organism [median of 358 (IQR 127, 800) WBC/mm^3^ for *N. meningitides* vs. 436 (99, 380) WBC/mm^3^ for *S. pneumoniae* vs. 1 (0, 2) WBC/mm^3^ for *Salmonella* sp., vs. 100 (100, 180) WBC/mm^3^ for culture-negative meningitis, *p* = 0.007]. Opening pressure was highest in children with meningococcal meningitis [median of 46 (IQR 32, 51) mmHg, 20 (14, 25) mmHg for *S. pneumoniae*, 15 (6, 19) mmHg for *Salmonella* sp. and 16 (12, 19) for culture-negative meningitis, *p* = 0.03]. No other variables were different when evaluated by infecting organism. Children with *Salmonella* sp. meningitis had better PCPC at discharge than children infected with other organisms [PCPC 1 (1, 2) for *Salmonella* sp., 3 (1, 4) for *N. meningitides*, 5 (3, 6) for *S. pneumoniae*, and 5 (4, 6) for culture-negative meningitis, *p* = 0.009].

**Table 1:** Demographics, vital signs, laboratory evaluations of children with bacterial meningitis (*n* = 47).

**Parameter**	**Value**	
Age (median, IQR)	45	(20, 115)
Female (total, %)	23	49
Convulsions prior to arrival (total, %)	28	61
Temperature, °C (mean, SD)	38.3	1.2
Heart rate, beats/min (mean, SD)	135	25
Respiratory rate, breaths/min (mean, SD)	36	13
Oxygen saturation, % (median, IQR)	97	(94, 98)
Mean BP, mmHg (median, IQR)	76	(61, 87)
Admission BCS (median, IQR)	1	(1, 2)
0 (*n*, %)	3	6
1 (*n*, %)	24	50
2 (*n*, %)	17	39
3 (*n*, %)	3	6
WBC, × 10^−9^/L (median, IQR)	7.2	(6.5, 15.9)
Packed cell volume, % (median, IQR)	28.5	(25.5, 34)
Glucose, mmol/L (mean, SD)	107	53
Lactate, mmol/L (median, IQR)	3.6	(1.8, 4.9)
Sodium, mmol/L (median, IQR)	139	(134, 142)
Bicarbonate, mEq/L (median, IQR)	19	(15, 21)
OP on LP, mmHg (median, IQR)	19	(13.5, 24.5)
CSF protein, mg/dL (median, IQR)	210	(100, 300)
CSF cells, WBC/mm^3^ (median, IQR)	247	(64, 400)
Organism (*n*, %)		
*S. pneumoniae*	17	36
*N. meningitides*	17	36
*Salmonella* sp.	6	13
Culture negative	5	11
Other organism	2	4
Discharge outcome (median, IQR)	2	(1, 4)
Good (*n*, %)	20	42
Poor (*n*, %)	27	58

*Min, minute; BP, blood pressure; WBC, white blood cells; BCS, Blantyre coma score; OP, opening pressure; LP, lumbar puncture; CSF, cerebrospinal fluid; Neg, negative*.

**Figure 2:**
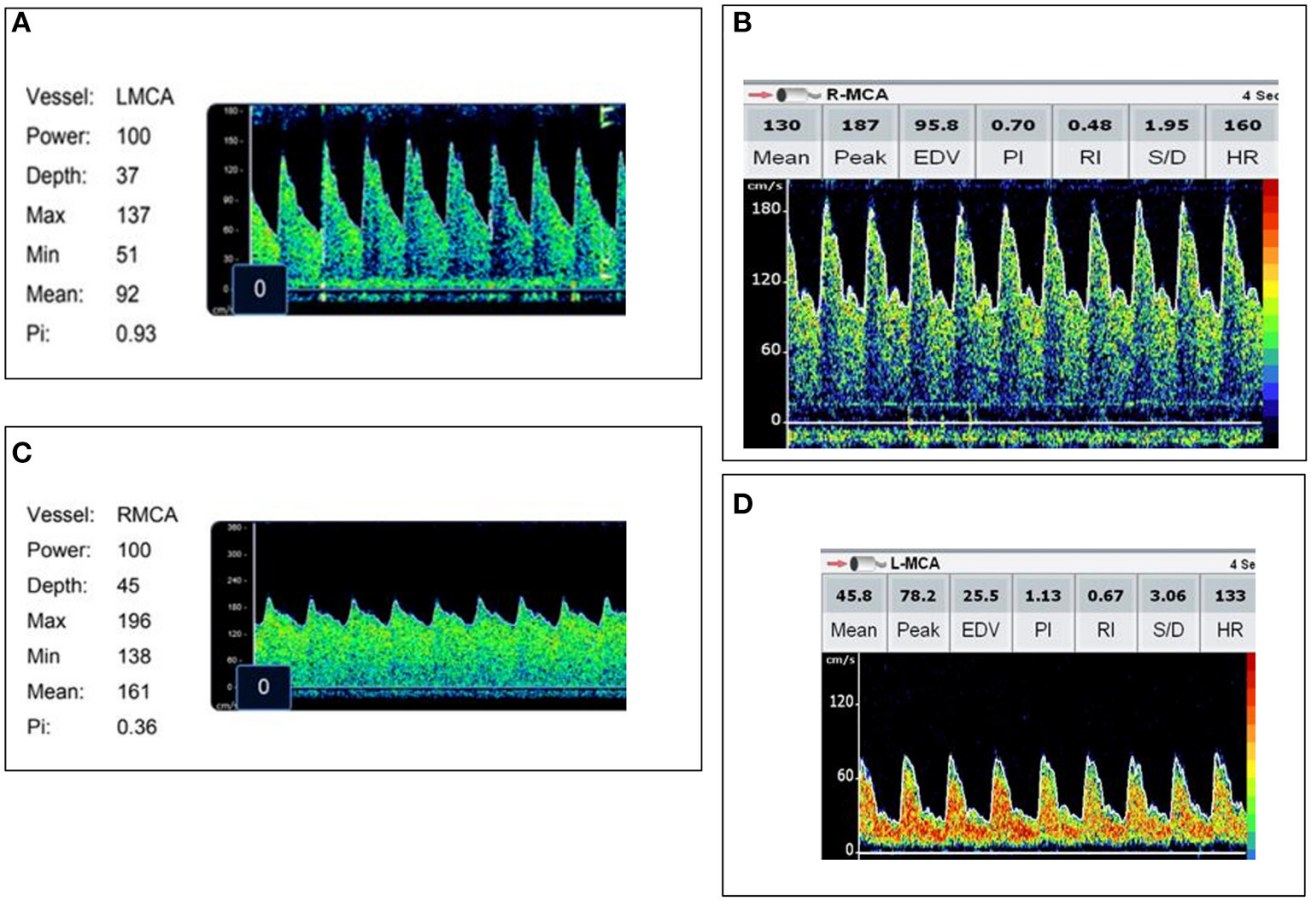
Representative images of each transcranial Doppler ultrasound (TCD) flow pattern. **(A)** Normal flow—Vs, Vd, Vm within 2SD of age normative value. **(B)** High flow/normal PI—Vs, Vm >2SD above age normal value and PI ≥0.6 and ≤1.3. **(C)** High flow/low PI—Vs, Vm >2SD above age normal value with a PI ≤0.5. **(D)** Low flow—Vs, Vm <2SD below age normal value.

Admission TCD was normal in 10/47 (21%) children. High flow with a normal PI was noted in 20/47 (43%) and high flow with a low PI was noted in 7/47 (15%). Ten of 47 (21%) children met criteria for low flow. Representative images of each flow pattern are in Figure 2. In four patients with low flow and normal PI on admission, the PI increased to >1.3 in eight TCD examinations. The increase in PI was identified on post-admission day 2 (IQR 1, 3). Demographic, vital sign, and laboratory variable differences between TCD flow types are in Table 2. Infection with a specific organism was not statistically associated with increased likelihood of the identification of any one flow pattern (*p* = 0.2).

**Table 2:** Patient characteristics by Transcranial Doppler ultrasonography flow pattern.

	**Normal (*****n*** **=** **10)**		**High flow, normal PI (*****n*** **=** **20)**		**High flow, low PI (*****n*** **=** **7)**		**Low flow (*****n*** **=** **10)**		*p*-value^*^
Age (median, IQR)	50	(14, 103)	59	(19, 133)	63	(36, 83)	72	(30, 96)	0.97
Convulsions prior to arrival (n, %)	4	40	11	55	2	29	7	70	0.14
Temperature, °C (mean, SD)	38.3	1.2	38.3	1.3	37.7	1.3	38.2	0.9	0.85
Heart rate, beats/min (mean, SD)	140	22	133	24	127	21	138	33	0.79
RR, breaths/min (mean SD)	35	8	36	12	36	12	32	13	0.88
Oxygen saturation, % (median, IQR)	98	(97, 99)	97	(93, 98)	96	(95, 98)	96	(93, 99)	0.30
Mean BP, mmHg (median, IQR)	67	(57, 86)	87	(55, 93)	79	(76, 83)	78	(78, 79)	0.66
Admission BCS (median, IQR)	2	(1, 2)	1	(1, 2)	2	(1, 2)	1	(0, 1)	0.62
WBC, × 10^−9^/L (median, IQR)	7.3	(5, 11)	9	(6, 21)	11	(8.5, 26)	8.2	(6.2, 10.2)	0.23
Packed cell volume, % (median, IQR)	32	(29, 36)	26	(21, 30)	29	(28, 35)	19	(18, 21)	0.17
Glucose, mmol/L (mean, SD)	90	(84, 101)	117	(114, 119)	114	(112, 117).	131	(122, 177)	0.05
Lactate, mmol/L (median, IQR)	3.3	(2.1, 6.6)	3.7	(1.7, 5)	2.3	(2, 2.9)	3.7	(2.1, 3.6)	0.83
Sodium, mmol/L (median, IQR)	139	(136, 142)	141	(139, 150)	134	(130, 136)	144	(133, 147)	0.56
Bicarbonate, mEq/L (median, IQR)	19	(13, 21)	16	(12, 21)	21	(20, 24)	27	(18, 29)	0.33
OP on LP, mmHg (median, IQR)	19	(13, 21)	17	(14, 23)	23	(20, 24)	17	(6.5, 38)	0.68
CSF Protein, mg/dL (median, IQR)	140	(100, 290)	240	(100, 300)	180	(100, 750)	300	(100, 390)	0.63
CSF cells, WBC/mm^3^ (median, IQR)	69	(3, 500)	140	(80, 360)	800	(100, 1500)	512	(54, 736)	0.32
Lindegaard ratio (mean, SD)	2	0.7	2.5	1.1	3.8	1.5	1.7	0.69	0.03
Autoregulation (median, IQR)	1.15	(1, 1.1)	1.06	(1.02, 1.09)	0.93	(0.46, 1.09)	1.09	(1.09, 1.13)	0.01
Outcome by PCPC (median, IQR)	1	(1, 2)	3	(1, 6)	5	(3, 5.5)	6	(2.5, 6)	0.007
Good									
1 (*n*, %)	8	80	5	25	–	–	1	10	
2 (*n*, %)	2	20	3	15	–	–	–	–	
Poor									
3 (*n*, %)	–	–	2	10	1	14	1	10	
4 (*n*, %)	–	–	3	15	–	–	1	10	
5 (*n*, %)	–	–	2	10	5	72	1	10	
6 (*n*, %)	–	–	5	25	1	14	6	60	

* *p values comparing “normal” to other groups*.

*PI, pulsatility index; n, number; Min, minute; RR, respiratory rate; BP, blood pressure; WBC, white blood cells; BCS, Blantyre coma score; OP, opening pressure; LP, lumbar puncture; CSF, cerebrospinal fluid; PCPC, pediatric cerebral performance category*.

When excluding children who died before the resolution of abnormal CBFVs, time to normalization varied between groups. All children with an initial normal TCD remained normal on subsequent examinations. Children with high flow/normal PI normalized after a median of 4 days (IQR 3, 6). Children with high flow/low PI normalized at a median of 10 days (IQR 4, 19). One patient with this flow pattern had persistently abnormal flows at discharge on day 40. Evaluation of the duration of abnormality in the low flow group was limited by the high mortality rate, but in survivors (*n* = 4), the median duration to normalization was 4 days (IQR 3, 5).

**Figure 3:**
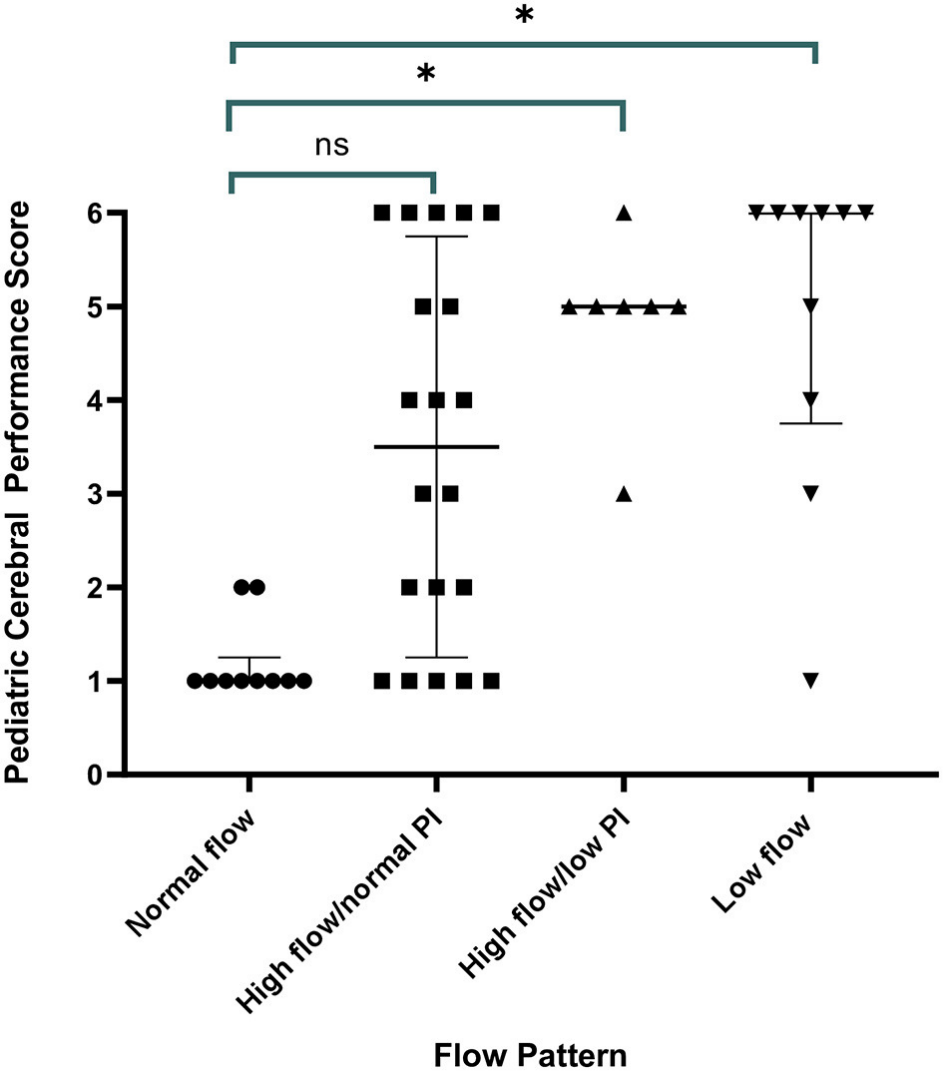
Discharge outcome by Pediatric Cerebral Performance Category (PCPC) by transcranial Doppler ultrasound (TCD) flow pattern. All children with a normal TCD flow pattern had a good neurological outcome. Children with high flow/normal PI had a median PCPC score of 3 (IQR 1, 6) (*p* = 0.11 vs. normal), children with high flow/low PI had a median PCPC score of 5 (IQR 3, 5.5) (*p* = 0.02 vs. normal), and children with low flow had a median PCPC score of 6 (IQR 2.5, 6) (*p* = 0.01 vs. normal).

All children with a normal TCD flow pattern had a good neurological outcome [median PCPC score of 1 (IQR 1, 2)]. PCPC was worse for patients with an abnormal CBFV pattern when compared with those with normal CBFVs (Table 2). If categorizing CBFV patterns as low risk (normal flow or high flow/normal PI) vs. high risk (high flow/low PI or low flow), patients with a high-risk pattern were more likely to have a poor outcome (82 vs. 38%, *p* = 0.001) (Figure 3). Poor outcome in survivors was uniformly spastic hemi- or quadriplegia consistent with multifocal infarct. There were no other significant clinical or laboratory differences between children who did well-compared with those who did poorly (Table 3).

**Table 3:** Differences in characteristics between children with a good vs. poor outcome.

**Characteristic**	**Poor outcome**		**Good outcome**		***p*-value**
Age (median, IQR)	115	(19, 144)	71.5	(24.5, 119)	0.55
Female (*n*, %)	4	44	3	38	>0.99
Temp, °C (mean SD)	38.73	0.91	37.75	1.58	0.13
HR, beats/min (mean SD)	135.67	27.68	127.25	20.89	0.49
RR, breaths/min (mean SD)	38.11	14.97	31.88	6.60	0.35
O2 sat, % (median, IQR)	96	(94, 97)	97	(93.5, 98)	0.77
Mean BP, mmHg (median, IQR)	93	(61, 94)	93.5	(61, 95)	>0.99
BCS (median, IQR)	1	(1, 2)	1.5	(1, 2)	>0.99
0 (*n*, %)	0	0	0	0	
1 (*n*, %)	2	67	1	50	
2 (*n*, %)	1	33	1	50	
3 (*n*, %)	0	0	0	0	
WBC, × 10^−9^/L (median, IQR)	13	(7, 24)	7.5	(5.7, 12.75)	0.39
PCV, % (median, IQR)	30	(26, 34)	23.5	(16, 25)	0.08
Glucose, mmol/L (mean, SD)	120.67	26.99	113.00	29.52	0.65
Lactate, mmol/L (median, IQR)	4.3	(2, 5.3)	1.85	(1.4, 4.8)	0.17
Na, mmol/L (median, IQR)	139	(129, 150)	141	(131, 149)	0.98
Bicarbonate, meq/L (median, IQR)	21	(12, 21)	17	(11, 20)	0.76
Convulsions on admission (*n*, %)	6	67	4	50	0.64
OP on LP, cmH20 (median, IQR)	16	(13, 23)	22	(18, 26)	0.66
CSF Protein, mg/dL (median, IQR)	270	(170, 315)	100	(100, 300)	0.35
CSF Cell count, WBC/mm^3^ (median, IQR)	178	(80, 360)	140	(0, 800)	0.94
Autoregulation index	1.07	(0.97, 1.2)	1.04	(1.02, 1.07)	0.94

*n, number; temp, temperature; HR, heart rate; Min, minute; RR, respiratory rate; O_2_ sat, oxygen saturation; BP, blood pressure; BCS, Blantyre coma score; WBC, white blood cells; PCV, packed cell volume; Na, sodium; OP, opening pressure; LP, lumbar puncture; CSF, cerebrospinal fluid*.

There was notable heterogeneity in the outcomes of children with high flow/normal PI. There were no differences in the demographics, vital signs, or laboratory parameters of children with a good vs. poor outcome with this TCD pattern. While not significant, children with good outcome were less febrile (mean temperature 37 ± 1.5°C vs. 38.9 ± 0.7°C), had lower peripheral WBC counts [7 (6, 9)·10^−9^/L vs. 17 (7, 24)·10^−9^/L], had lower lactate [1.8 (1.4, 5) mmol/L vs. 4.3 (2, 4.9) mmol/L], and lower CSF WBC counts [75 (0, 800) vs. 239 (140, 360) WBC/mm^3^]. When evaluating the LR, PI, and THRR in children with this flow pattern, LR was not significantly different between children with good (2.24 ± 0.75) vs. poor outcome (2.3 ± 0.66) (*p* = 0.88). PI was lower in children with a good outcome (0.73 ± 0.19) than in those with a poor outcome (0.97 ± 0.31, *p* = 0.01). Autoregulation was significantly better in children with a good outcome (THRR1.12 ± 0.06) compared with those with a poor outcome (0.98 ± 0.05) (*p* = 0.03).

## Discussion

BM is an important cause of long-term neurodisability and mortality in developing countries (1–8). Beyond the provision of effective, timely antibiotics, other adjunctive treatments that have been evaluated in these regions of the world have not had an impact on outcomes (32–38). Therapy directed at improving CPP in patients with CNS infection has shown significant promise in improving long-term functional status in other settings (14–16). Thus, finding a means by which to identify high-risk children with BM in sub-Saharan Africa who would benefit from CPP augmentation may be a first step in reducing the impact of this disease.

TCD is a portable, non-invasive tool that evaluates the cerebrovascular hemodynamics (18–20, 39–42). We performed this study to evaluate the CBFVs in children with BM and found (1) 21% of children had normal admission CBFVs and ALL of them had good neurologic outcome, (2) 40% of children had TCD evidence of cerebral hypoperfusion (high CBFV/low PI or low CBFV) and the majority of them had poor outcomes (14/17, 82%), and (3) the remaining children had elevated CBFVs with normal PI and variable outcomes.

Three previous studies report the use of TCD to evaluate the cerebral vasculature in children with BM outside the neonatal period (22–24). One did not compare the CBFVs in the children with BM with either control patients or normative values for age, making the interpretation of the results difficult (23). Bode et al. reported a 130–150% increase in MCA Vm in 11/15 (73%) of children with BM compared with normative values (22). The children with BM in this work were part of a larger cohort of children affected by various neurological illnesses beyond CNS infection. Outcomes were not separated by etiology of illness in the analysis, making interpretation of the impact of abnormal CBFVs in children with BM difficult. Ducharme et al. reported TCD findings in a cohort of children with mixed etiologies of CNS infection (24). Ten children with BM had TCD performed in the acute phase of illness (one child with BM had TCD examination only after day 30). Two children with BM had a normal TCD (compared with age normative value), seven had increased CBFVs, and one had low CBFVs. Across the entire cohort, the presence of hypoperfusion in at least one vessel (Vm >120 cm/s with LR >3 or absolute Vm >200 cm/s OR low flow <1SD from age normal) was associated with acquired ICU morbidity (*p* = 0.04) and death (*p* = 0.03). Our findings are similar to these previously reported works, but in a much larger, distinct patient population.

For the first time, we also identified clear differences in neurological outcomes associated with different CBFV patterns in BM. Children in our study with normal CBFVs likely represented individuals with no or minimal neurovascular involvement that fully recovered with antibiotics and supportive care. CBFVs are elevated when large vessel narrowing occurs. Children with high CBFVs and a normal PI were found to have significant variability in outcome. While not statistically significant, there were differences in vital signs and laboratory parameters suggesting the children with good outcomes were less severely ill. There was no difference in the LR between those with a good vs. poor outcome, suggesting the degree of vessel obstruction was not different. Rather, TCD measurements revealed that the PI was lower and autoregulation was more intact in children with good outcome. This suggests that perhaps some children in this group had better outcomes because they were able to sufficiently augment flow to meet demand via peripheral vasodilation.

An extremely low PI in the setting of elevated CBFVs represents clear evidence of impaired distal perfusion beyond a point of flow obstruction. The LR was most significantly elevated in these children (3.8 ± 1.5, *p* = 0.03 vs. normal flow), suggestive of significant vessel diameter reduction. In this instance, distal vessels maximally vasodilate to reduce resistance to flow. This results in increased flow during diastole and a low measured PI. However, as the obstruction is relatively fixed in BM, flow may not sufficiently increase to meet demand despite peripheral vasodilation, and secondary ischemic injury may occur. This likely explains the significant morbidity in these children. Children with high CBFVs and low PI may be those who would most benefit from adjunctive interventions to augment CPP. Future studies could be designed to answer this question.

We identified a significant proportion of children with low CBFVs. Investigative studies of progressive intracranial vascular occlusion in other diseases outline a “tipping point” of vessel lumen diameter reduction where flow becomes so impaired that measured CBFVs transition from elevated to reduced (27, 28). Animal studies of BM have identified cortical blood flow initially increases but then decreases to <30% of baseline values as illness severity worsens (43). Autopsy studies in humans have also revealed that progressive inflammation in BM can result in malignant arteritis obliterans (44). Progressive narrowing may have contributed to the TCD findings of low flow in our study. Insufficient flow would result in ischemic injury and subsequent intracranial hypertension if cerebral edema ensued. In support of this consideration, poor outcome was nearly uniform in these children. In addition, only four children had TCD studies where an increased PI >1.3 was identified and all were in the low flow group. This finding is strongly suggestive of increased CVR that is most consistent with intracranial hypertension in this clinical scenario. Due to poverty, lack of reasonably distanced health centers, and many other factors, late presentation to care is common in resource-limited settings (45, 46). The high proportion of children identified with low CBFVs compared with other previous reports from other settings may be reflective of advanced disease at the time of admission. It remains unclear if these children would have benefit if attempts to augment CPP would be made.

Overall, while done in a resource-limited setting in Africa, the findings of this study should be applicable to the care of children in any setting. TCD is portable, widely available in centers that care for pediatric patients, and can be performed repeatedly to evaluate the cerebral hemodynamics of children with BM. If a high-risk flow pattern is identified, practitioners could consider further neuroimaging, further investigation with multi-modal neuromonitoring, and potentially CPP augmentation.

### Limitations

The relatively small sample size limited the number of children identified with each CBFV pattern. However, the work does represent the largest cohort of children with BM evaluated by TCD to date. Even with small sample sizes, there were significant differences in the PCPC scores between groups. Thus, it is likely that these associations would continue to hold true with a larger sample size. Another limitation is that normative values for TCD measurements do not exist for African children. As such, the results of the TCD studies were compared with previously published pediatric normative values, which were largely Caucasian children. Future studies are needed to ensure appropriate normative values are developed for this patient population given the possible alterations in expected flow velocities based on race and common comorbidities. Lastly, neuroimaging was not available, and thus correlations between clinical findings concerning for neurologic impairment, TCD measurements, and abnormal findings on CT or MRI were not possible.

## Conclusions

TCD can be used to non-invasively evaluate the neurovasculature of children with BM and identify those at high risk of poor outcome.

## Data Availability Statement

The original contributions presented in the study are included in the article/supplementary materials, further inquiries can be directed to the corresponding author.

## Ethics Statement

The studies involving human participants were reviewed and approved by Kinshasa School of Public Health Ethics Committee and the Malawi College of Medicine Ethics Committee. Written informed consent to participate in this study was provided by the participants' legal guardian/next of kin.

## Author Contributions

All authors participating in study design, interpretation of the data, and manuscript preparation. YF, TT, JP, MU, and NO'B participated in TCD examination performance.

## Conflict of Interest

The authors declare that the research was conducted in the absence of any commercial or financial relationships that could be construed as a potential conflict of interest.

## References

[1] World Health Organization (WHO). Meningococcal Meningitis: Fact Sheet 2017. (2017). Available online at: http://www.who.int/mediacentre/factsheets/fs141/en/ (accessed September 30, 2019).

[2] Murray CJL Vos T Lozano R Naghavi M Flaxman AD Michaud C. Disability-adjusted life years (DALYS) for 291 diseases and injuries in 21 regions, 1990-2010: a systemic analysis for the Global Burden of Disease Study. Lancet. (2012) 380:2197–223. 10.10161/S0140-6736(12)61689-4 23245608

[3] Swartz MN. Bacterial meningitis—a view of the past 90 years. N Engl J Med. (2004) 351:1826–8. 10.1056/NEJMp048246 15509815

[4] Stephens DS Greenwood B Brandtzaeg P. Epidemic meningitis, meningococcaemia, and Neisseria meningitidis. Lancet. (2007) 369:2196–210. 10.1016/S0140-6736(07)61016-2 17604802

[5] Chandran A Herbert H Misurski D Santosham M. Long-term sequelae of childhood bacterial meningitis: an underappreciated problem. Pediatr Infect Dis J. (2011) 30:3–6. 10.1097/INF.0b013e3181ef25f7 20683377

[6] Edmond K Clark A Korczak VS Sanderson C Griffiths UK Rudan I. Global and regional risk of disabling sequelae from bacterial meningitis: a systematic review and meta-analysis. Lancet Infect Dis. (2010) 10:317–28. 10.1016/S1473-3099(10)70048-7 20417414

[7] Sheld W Michael Koedel U Nathan B Hans-Walter P. Pathophysiology of bacterial meningitis: mechanisms of neuronal injury. J Inf Dis. (2003) 186:225–33. 10.1086/344939 12424702

[8] Wright JP Ford HL. Bacterial meningitis in developing countries. Trop Doct. (1995) 25:5–8. 10.1177/004947559502500102 7886841

[9] Muller M Merkelbach S Huss GP Schimrigk K. Clinical relevance and frequency of transient stenosis of the middle and anterior cerebral arteries in bacterial meningitis. Stroke. (1995) 26:1399–403. 10.1161/01.STR.26.8.1399 7631344

[10] Hans-Walter P Gian DB Ulrich D Bauer M Einhaupl KM. Cerebrovascular complications of bacterial meningitis in adults. Neurology. (1992) 42:8–16. 10.1212/WNL.42.8.1497 1641143

[11] Klein M Koedel U Pfefferkorn T Zeller G Woehrl B Hans-Walter P. Arterial cerebrovascular complications in 94 adults with acute bacterial meningitis. Crit Care. (2011) 15: R281. 10.1186/cc10565 22112693 PMC3388646

[12] Schut ES Lucas MJ Brouwer MC Vergouwen MDI van der Ende A van de Beek D. Cerebral infarction in adults with bacterial meningitis. Neurocrit Care. (2011) 16:421–7. 10.1007/s12028-011-9634-4 21989842

[13] Kastenbauer S Pfister HW. Pneumococcal meningitis in adults: spectrum of complications and prognostic factors in a series of 87 cases. Brain. (2003) 126:1015–25. 10.1093/brain/awg113 12690042

[14] Ries S Schminke U Fassbender K Daffertshofer M Steinke W Hennerici M. Cerebrovascular involvement in the acute phase of bacterial meningitis. J Neurol. (1997) 244:51–5. 10.1007/s004150050050 9007746

[15] Sigmon JL Ball AM Tucker KL. Treatment of vasospasm in bacterial meningitis with hemodynamic augmentation. Neurol Dis Ther. (2017) 1:1–5. 10.15761/NDT.1000122

[16] Moller K Larsen FS Qvist J Wandall JH Knudsen GM Gjorup IE. Dependency of cerebral blood flow on mean arterial pressure in patients with acute bacterial meningitis. Crit Care Med. (2000) 28:1027–32. 10.1097/00003246-200004000-00019 10809277

[17] Kumar R Singhi S Singhi P Jayashree M Bansal A Bhatti A. Randomized controlled trial comparing cerebral perfusion pressure-targeted therapy versus intracranial pressure-targeted therapy for raised intracranial pressure due to acute CNS infections in children. Crit Care Med. (2014) 42:1775–87. 10.1097/CCM.0000000000000298 24690571

[18] Aaslid R Markwalder TM Nornes H. Noninvasive transcranial Doppler ultrasound recording of flow velocity in basal cerebral arteries. J Neurosurg. (1982) 57:769–74. 10.3171/jns.1982.57.6.0769 7143059

[19] Lindegaard KF Nornes H Bakke SJ Sorteberg W Nakstad P. Cerebral vasospasm diagnosis by means of angiography and blood velocity measurements. Acta Neurochir. (1989) 100:12–24. 10.1007/BF01405268 2683600

[20] Bode H Wais U. Age dependence of flow velocities in basal cerebral arteries. Arch Dis Childhood. (1988) 63:606–11. 10.1136/adc.63.6.606 3389890 PMC1778883

[21] Koc RK Akdemir H Menku A. Evaluation of Cerebral Blood Flow Velocity with transcranial doppler ultrasound in central nervous system infections. Turkish Neurol. (1998) 8:76–81.

[22] Bode H Harders A. Transient stenosis and occlusion of main cerebral arteries in children- diagnosis and control of therapy by transcranial Doppler sonography. Eur J Pediatr. (1989) 148:406–411. 10.1007/BF00595898 2646127

[23] Okten A Ahmetoglu A Dilber E Dinc H Kalyoncu M Ciftcibais K. Cranial Doppler ultrasonography as a predictor of neurologic sequelae in infants with bacterial meningitis. Investigative Radiol. (2002) 37:86–90. 10.1097/00004424-200202000-00006 11799332

[24] Ducharme-Crevier L Mills M Mehta P Smith CM Wainwright M. Use of transcranial Doppler for management of central nervous system infections in critically ill children. Ped Neurol. (2016) 65:52–8. 10.1016/j.pediatrneurol.2016.08.027 27743745

[25] Goh D Minns R. Cerebral blood flow velocity monitoring in pyogenic meningitis. Arch Dis in Childhood. (1993) 68:111–9. 10.1136/adc.68.1.111 8434994 PMC1029196

[26] Rosin IA. Doppler ultrasonography in children with sequelae of acute neuroinfections. Zh Nevrol Psik. (1999) 99:23–5. 10578530

[27] Spencer MP Reid JM. Quantitation of carotid stenosis with continuous wave Doppler ultrasound. Stroke. (1979) 10:326–30. 10.1161/01.STR.10.3.326 462521

[28] Alexandrov A. The Spencer's curve: clinical implications of a classic hemodynamic model. J Neuroimaging. (2007) 17:6–10. 10.1111/j.1552-6569.2006.00083.x 17238866

[29] Smielewski P Czosnyka M Kirkpatrick P McEroy H Rutkowska H Pickard JD. Assessment of cerebral autoregulation using carotid artery compression. Stroke. (1996) 27:2197–203. 10.1161/01.STR.27.12.2197 8969780

[30] Fiser DH. Assessing the outcome of pediatric intensive care. J Pediatr. (1992) 121:68–74. 10.1016/S0022-3476(05)82544-2 1625096

[31] Fiser DH Tilford JM Roberson PK. Relationship of illness severity and length of stay to functional outcomes in the pediatric intensive care unit: a multi-institutional study. Crit Care Med. (2000) 28:1173–9. 10.1097/00003246-200004000-00043 10809301

[32] Odio CM Faingezicht I Paris M Nassar M Baltodano A Rogers J. The beneficial effects of early dexamethasone administration in infants and children with bacterial meningitis. N Engl J Med. (1991) 324:1525–31. 10.1056/NEJM199105303242201 2027357

[33] van de Beek D Farrar JJ de Gans J Nguyen THM Molyneux EM Peltola H. Adjunctive dexamethasone in bacterial meningitis: a meta-analysis of individual patient data. Lancet Neurol. (2010) 9:254–63. 10.1016/S1474-4422(10)70023-5 20138011 PMC2835871

[34] Borchorst S Møller K. The role of dexamethasone in the treatment of bacterial meningitis - a systematic review. Acta Anaesthesiol Scand. (2012) 56:1210–21. 10.1111/j.1399-6576.2012.02698.x 22524556

[35] Peltola H Roine I Fernández J Zavala I Gonzalez Ayala S Gonzalez Mata A. Adjuvant glycerol and/or dexamethasone to improve the outcomes of childhood bacterial meningitis: a prospective, randomized, double-blind, placebo-controlled trial. Clin Infect Dis. (2007) 45:1277–86. 10.1086/522534 17968821

[36] Kilpi T Peltola H Jauhiainen T Kallio MJ. Oral glycerol and intravenous dexamethasone in preventing neurologic and audiologic sequelae of childhood bacterial meningitis. The Finnish Study Group. Ped Infect Dis J. (1995) 14:270–8. 10.1097/00006454-199504000-00005 7603807

[37] Peltola H Leib S. Performance of adjunctive therapy in bacterial meningitis depends on circumstances. Ped Infect Dis J. (2013) 32:1381–2. 10.1097/INF.0000000000000066 24569310

[38] Ajdukiewicz K Cartwright KE Scarborough M Mwambene JB Goodson P Molyneux ME. Glycerol adjuvant therapy in adults with bacterial meningitis in a high HIV seroprevalence setting in Malawi: a double-blind, randomised controlled trial. Lancet Infect Dis. (2011) 11:293–300. 10.1016/S1473-3099(10)70317-0 21334262 PMC3999512

[39] O'Brien NF Mutatshi Taty T Moore-Clingenpeel M Bodi Mabiala J Pongo J Ambitapio Musungufu D. Transcranial Doppler ultrasonography provides insights into neurovascular changes in children with cerebral malaria. J Pediatr. (2018) 203:116–24. 10.1016/j.jpeds.2018.07.075 30224088

[40] Lagunju I Sodeinde O Telfer P. Prevalence of transcranial Doppler abnormalities in Nigerian children with sickle cell disease. Am J Hematol. (2012) 87:544–7. 10.1002/ajh.23152 22460323

[41] Kija EN Saunders DE Munubhi E Darekar A Barker S Cox TCS. Transcranial Doppler and magnetic resonance in Tanzanian children with sickle cell disease. Stroke. (2019) 50:1719–26. 10.1161/STROKEAHA.118.018920 31195937 PMC6594727

[42] Hokazono M Silva GS Braga JA. Results from transcranial Doppler examination on children and adolescents with sickle cell disease and correlation between the time-averaged maximum mean velocity and hematological characteristics: a cross-sectional analytical study. São Paulo Med J. (2011) 129:134–8. 10.1590/S1516-31802011000300003 21755247 PMC10866317

[43] Tauber MG. Brain edema, intracranial pressure, and cerebral blood flow in bacterial meningitis. Ped Infect Dis J. (1989) 8:915–7. 10.1097/00006454-198912000-00042 2696929

[44] Mook-Kanamori BB Geldhoff M van der Poll T van de Beek D. Pathogenesis and pathophysiology of pneumococcal meningitis. Clin Micro Rev. (2011) 24:557–91. 10.1128/CMR.00008-11 21734248 PMC3131058

[45] Ni P Yagou-Ide Le. Challenges in the management of sepsis in a resource poor setting. Int J Clin Med. (2017) 8:412–21. 10.4236/ijcm.2017.8603928349179

[46] Rose A. Late presentation to hospital services necessitates greater community-based care for malnourished children. J Trop Pediatr. (2015) 61:61–4. 10.1093/tropej/fmu059 25389182

